# Newborn Male With Fibular Aplasia, Tibial Campomelia, and Oligosyndactyly Syndrome: A New Case Report Putting the Condition Under Spotlight

**DOI:** 10.7759/cureus.21702

**Published:** 2022-01-28

**Authors:** Marian K Georgeos, Dina R Elgzzar

**Affiliations:** 1 Neonatal Intensive Care Unit, Ain Shams General Hospital, Cairo, EGY

**Keywords:** split hand/foot malformation, fatco syndrome, ectrodactyly, tibial campomelia, fibular hemimelia

## Abstract

The syndrome of fibular aplasia, tibial campomelia, and oligosyndactyly (FATCO syndrome) is a rare genetic disease that has been increasingly reported over the past 40 years. We report the case of a newborn boy with unilateral skeletal abnormalities that were evident clinically and radiologically. The baby was an infant of a diabetic mother, and the Egyptian parents were consanguineous with a strong family history of genetic diseases and congenital anomalies. Besides describing a new case report of this syndrome, we emphasize the importance of prenatal diagnosis and genetic counseling, especially for families at high risk for genetic diseases in developing countries.

## Introduction

The term “skeletal dysplasia” refers to diverse genetic disorders of the skeleton that manifest during prenatal and postnatal growth. Deformities of the limbs include hypoplasia and reduction defects. Limb deficiency is defined as the congenital absence or hypoplasia of a long bone, digits, or both. The prevalence of all types of limb deficiency is 0.79 per 1000 live births, of which the upper limb deficiencies occur more frequently than those of the lower limb. The most common apparent cause of limb deficiencies is vascular disruption defects (0.22 cases per 1000 live births), such as amniotic band-related limb deficiency. Limb deficiencies can be divided into the following two types: longitudinal and transverse deficiencies. Longitudinal bone deficiencies, which are more common, manifest themselves as an abnormal development (i.e., complete or partial absence) of the radius, fibula, or tibia. Transverse deficiencies are the complete absence of a whole limb or only a segment of it. Radial bone deficiency is the most common among upper extremity deficiencies, whereas fibular hypo- or aplasia is the most common of those of the lower limb [1-2].

Fibular hemimelia (also known as postaxial hypoplasia of the lower extremity), in contrast, is very rare, occurring in only 1 of 40,000 births. Disruptions during the critical period of embryonic limb development (fourth to seventh week of gestation) can cause varying degrees of fibular hypoplasia or aplasia [3]. We describe an infant with skeletal deformities typical of fibular aplasia, tibial campomelia, and oligosyndactyly (FATCO) syndrome.

## Case presentation

A male neonate was born at approximately 35 weeks of gestation by cesarean section to a mother who had premature contractions which led to premature rupture of membranes. Also, the mother had undergone a previous cesarean section. His Apgar scores were three in the first minute and seven in the fifth minute. The patient was admitted to our neonatal intensive care unit (NICU) soon after birth with grade II respiratory distress and skeletal deformities.

The mother was 34 years old, G5P3+1; she suffered from gestational diabetes mellitus, which was controlled by insulin, and she took vitamins and tocolytics for premature contractions but she did not receive any prophylactic antibiotics. Nothing else in her medical or obstetric history was remarkable. She had attended her prenatal visits regularly and had undergone prenatal ultrasonography repeatedly during her pregnancy. She had no history of antenatal steroid use. She and the baby’s father, who was 39 years old, were consanguineous (first cousins). They had a strong history of previous children with congenital anomalies; the first (boy) and fourth (girl) babies were normal and healthy, but the second (boy) had cerebral palsy of unknown cause and died at the age of seven years, and the third pregnancy was terminated in the fifth month because multiple congenital anomalies were discovered. Of more importance was that the parents mentioned that they had disregarded previous medical advice to receive genetic counseling.

**Figure 1:**
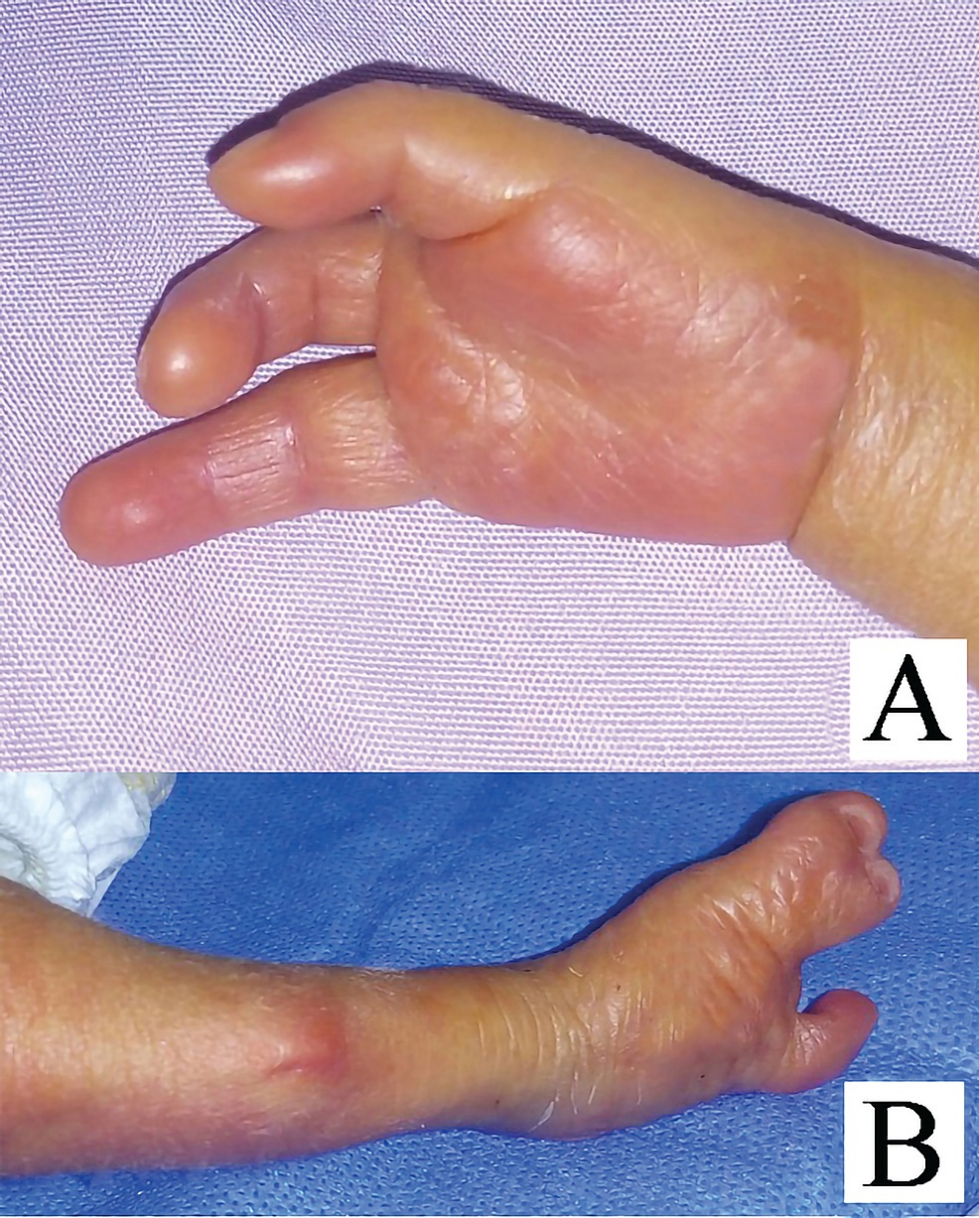
Clinical photographs of the patient. (A) Three-ray right hand: the thumb, the little finger, and one finger in between. (B) The right foot shows split hand/foot malformation, manifested as syndactyly between the two medial toes which were separated by a wide gap from the little toe on the lateral side.

**Figure 2:**
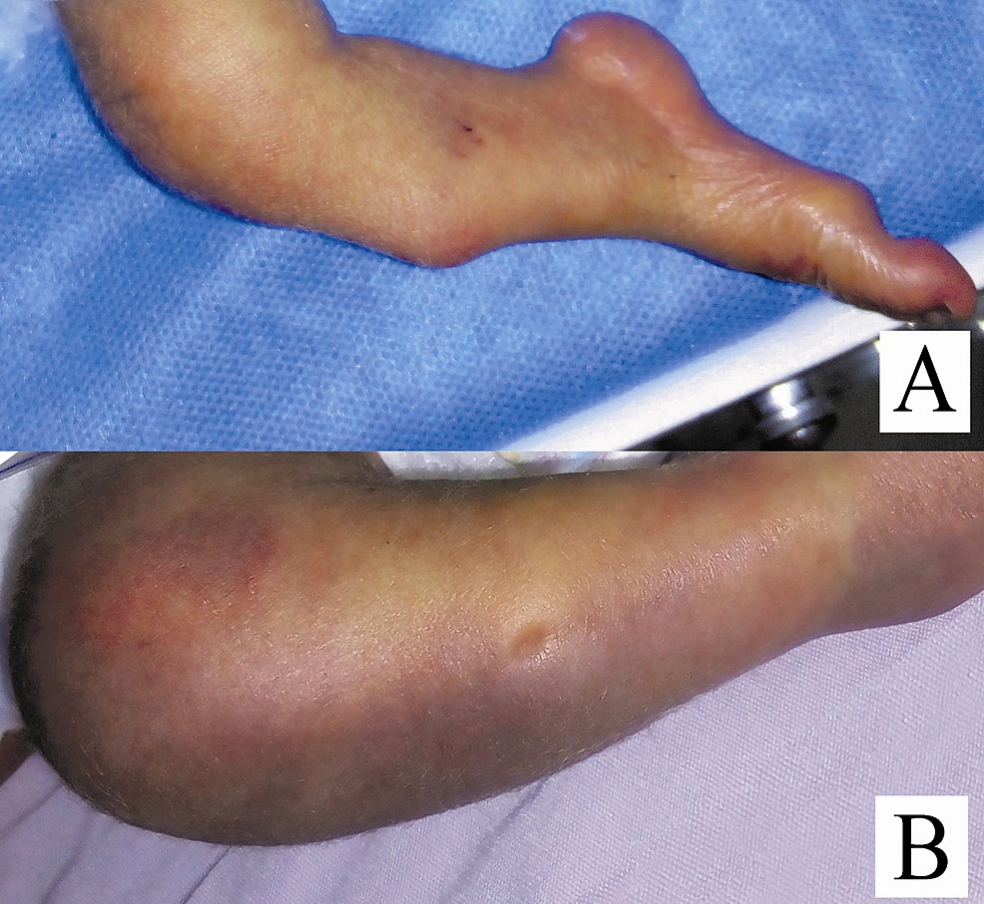
(A) Right tibial campomelia. (B) Skin dimple in the middle of the right tibia.

On admission, aside from being tachypneic (respiratory rate was 65 breaths/min), the baby’s vital signs were within normal ranges. According to growth charts, the birth weight (1875 g) was at the 10th percentile, the length (40 cm) was below the third percentile, and the head circumference (29.5 cm) was at the 10th percentile. Physical examination revealed brachycephaly, but no unusual facies, and multiple prominent skeletal deformities, including oligodactyly of the right hand, manifesting as only three fingers (the thumb, the little finger, and one finger in between), and a right lower limb that was shorter than the left lower limb because of abnormal angulation in the middle of the tibia, with overlying skin dimple. Another deformity was ectrodactyly in the right foot, manifesting as syndactyly between the two medial toes, which were separated from the little toe on the lateral side by a wide gap, characteristic of split hand/foot malformation (Figures 1A,B and 2A,B). The left upper and lower limbs were normal.

Chest examination revealed bilateral decreased air entry with mild intercostal and subcostal retractions. No abnormalities of the head, neck, heart, abdomen, or genitalia were discovered.

**Figure 3:**
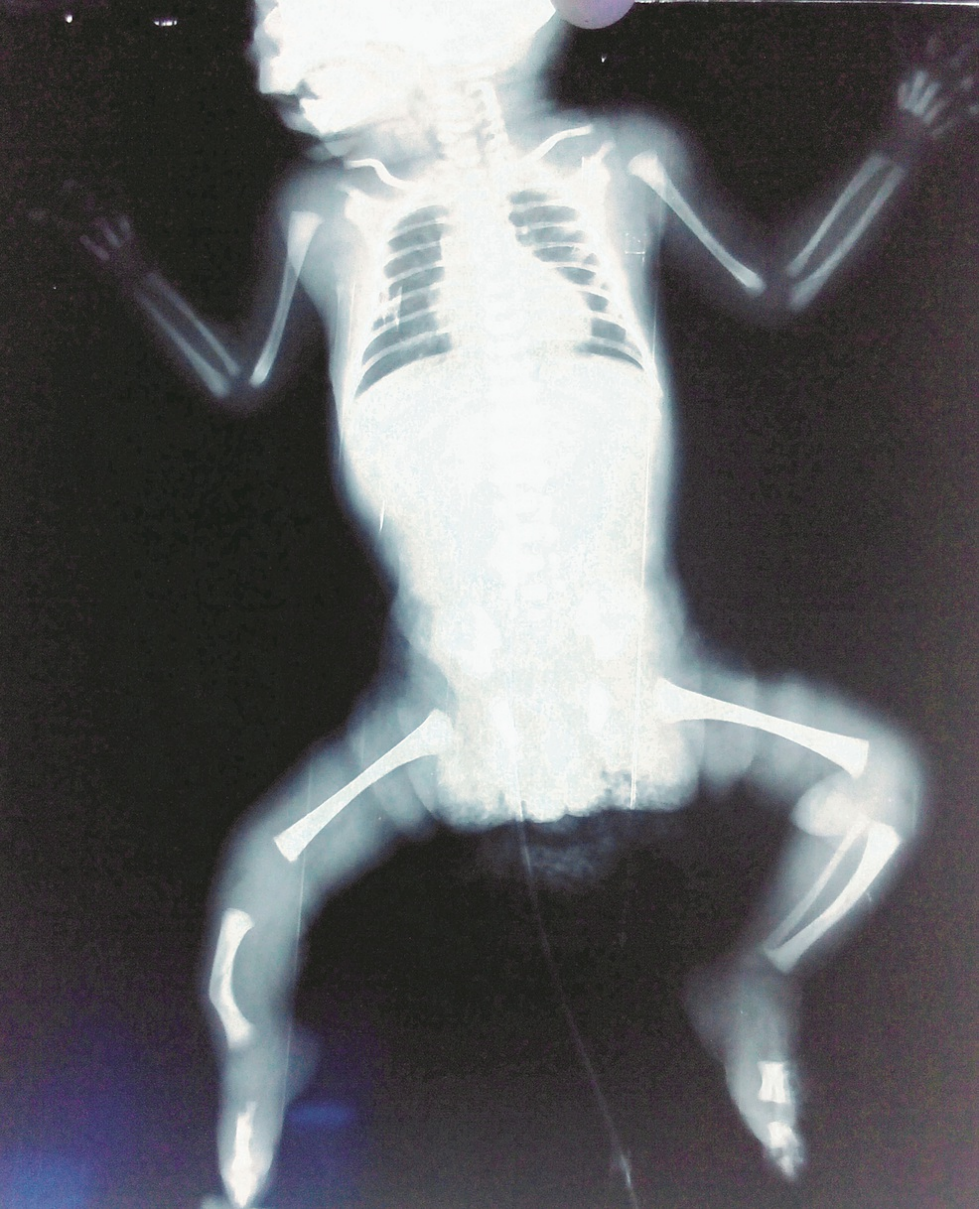
Radiograph of the patient. It shows the right hand with only three metacarpals, the normal left hand, right fibular hemimelia, middle tibial campomelia, and absent calcaneus and talus ossification centers and normal femurs, left tibia, and left fibula.

Radiographs showed the three-ray right hand and three-ray right foot with syndactyly between the first and second toes, right fibular hemimelia, and middle tibial campomelia. The left upper and lower limbs appeared normal. The femurs on both sides appeared normal; however, calcaneus and talus ossification centers were absent, which normally appear during the sixth and seventh months in utero, respectively (Figure 3).

Other investigations (e.g., echocardiography and cranial and abdominal ultrasonography) did not reveal any associated anomalies. No genetic study was performed because it was unavailable at our center. Unfortunately, on day three of life, the baby developed repeated apnea attacks, mostly because of early onset sepsis, as proved by the sepsis workup (e.g., complete blood count, C-reactive protein, and blood culture which revealed positive growth of group B Streptococci bacteria) done for the patient, for which he was ventilated; however, his condition continued to deteriorate. The baby died at the age of seven days from septicemia and disseminated intravascular coagulation.

## Discussion

Our patient represented the first case of pure FATCO syndrome to be reported in Egypt. A comparable case in Egypt in 2017 was reported as a combination of FATCO and Fuhrmann syndromes [4]. The literature contains very few other descriptions of FATCO syndrome; in addition to our patient, only 26 cases of FATCO syndrome have been published thus far, to our knowledge. Of interest is that of those 27 patients (whose gender was reported, including our case), only eight were female (male-to-female ratio, 3.4:1); hence, the condition has a male predominance. Both the upper and lower limbs were affected in 12 cases, whereas only the lower limbs were affected in 15 cases. Although only one side was affected in our patient, bilateral involvement was common in most of the previously reported cases.

In our patient, we found no associated anomalies in the other body systems. In contrast, of the two affected patients reported by Hecht and Scott [5], one (the male infant) had congenital cyanotic heart disease, and Bieganski et al. described one affected patient with associated membranous ventricular septal defect [6].

Our patient had no characteristic abnormal facies. In contrast, Kitaoka et al. reported an affected patient with cleft lip and palate [7]; Capece et al. described an affected patient with facial dysmorphia, manifested by a flat face, large ears, and ankyloblepharon [8]; Huber et al. described an affected patient with retrognathia and a crease in one earlobe [9].

No genetic study was performed in our patient because such testing was unavailable at our center. Despite many speculations being proposed, the explanation of the genetic basis of FATCO syndrome remains unrevealed. In the first case report published on FATCO syndrome, Hecht and Scott described the disorder in half-siblings whose mother was healthy. One of these was a boy with severe deformities involving the upper and lower limbs; agenesis of both hands, the left leg, and the right foot. Accordingly, the authors suggested that the genetic basis of the disease was an autosomal dominant mutant gene with decreased penetrance or gonadal mosaicism in the mother [5]. A faulty WNT7A gene was ruled out by Kitaoka et al. and Karaman and Kahveci as a probable cause of FATCO syndrome [7, 10]. In their study which was carried out on three patients with FATCO syndrome, Bieganski et al. searched for mutations in the TP63 and WNT10B genes, but they found no genetic abnormalities [6]. Bastaki et al. assumed that the majority of patients with FATCO syndrome have the phenotype of fibular agenesis with ectrodactyly. To verify this hypothesis, the molecular basis of FATCO syndrome needs to be investigated [11].

Because of the male predominance, some authors have suggested that FATCO syndrome is X-linked. If this is correct, it would explain why the manifestations were more severe in the male patient reported by Hecht and Scott than in his half-sister [5].

The plan of management is tailored according to the findings of each case. The aim of treatment for fibular hemimelia is reducing the length difference between the two legs and stabilization of the knee and ankle joints. The most popular methods of management include the use of an orthotic device, epiphysiodesis, limb lengthening, and amputation (followed by placement of a prosthesis), in addition, external circular fixators have been used for limb salvage and showed favorable outcomes [12].

In cases in which the foot is nonfunctional and the leg is more than 50% shorter at birth than expected, Syme or Boyd amputation should be performed, and use of a prosthesis should be instituted early [13]. Unfortunately, we could not apply these modalities of treatment in our patient.

## Conclusions

We report the 27th known case of FATCO syndrome, a rare genetic developmental disorder of the limbs whose genetic cause has not yet been identified. FATCO syndrome diagnosis during early prenatal stages remains challenging, especially in developing countries. Genetic counseling and discussions with the parents about this condition and its management are of utmost importance; we strongly recommend them particularly for families with a history of congenital anomalies.
